# Electrical Impedance Tomography of Electrolysis

**DOI:** 10.1371/journal.pone.0126332

**Published:** 2015-06-03

**Authors:** Arie Meir, Boris Rubinsky

**Affiliations:** 1 Biophysics Graduate Program, University of California, Berkeley, California, United States of America; 2 Department of Mechanical Engineering, University of California, Berkeley, California, United States of America; University of Houston, UNITED STATES

## Abstract

The primary goal of this study is to explore the hypothesis that changes in pH during electrolysis can be detected with Electrical Impedance Tomography (EIT). The study has relevance to real time control of minimally invasive surgery with electrolytic ablation. To investigate the hypothesis, we compare EIT reconstructed images to optical images acquired using pH-sensitive dyes embedded in a physiological saline agar gel phantom treated with electrolysis. We further demonstrate the biological relevance of our work using a bacterial *E*.*Coli* model, grown on the phantom. The results demonstrate the ability of EIT to image pH changes in a physiological saline phantom and show that these changes correlate with cell death in the E.coli model. The results are promising, and invite further experimental explorations.

## Introduction

Tissue ablation with minimally invasive surgery is important for treatment of many diseases and has an increasing role in treatment of solid neoplasms. A variety of biophysical and biochemical processes are used for this purpose. They include thermal ablation with heating, cooling or freezing, electroporation, injection of chemical agents, photodynamic effects, sonoporation effects and many others. Electrolysis, the passage of a low magnitude direct ionic current through the tissue, between two electrodes, is a biochemical/biophysical process that has been considered for tissue ablation since the 19^th^ century [1]. Electrolysis affects the ionic species in tissue, which change into compounds that can ablate cells. The advantage of electrolysis in comparison to other ablation techniques can be attributed to its simplicity and low cost of instrumentation, which might make it a suitable treatment modality for resource constrained communities where more expensive medical treatment is often not available [2].

The work of Nordenstrom and colleagues [3,4], is among the early modern work on electrolysis. Important recent work was published on understanding of the effects of electrolysis on tissue through histology, mathematical modeling of involved electrochemical processes, and clinical work, e.g. [5–15] and [16–19]. Several findings were made and several research techniques were developed that have inspired this paper: it was shown that the electrolysis induced pH changes can be used to reliably monitor the extent of tissue ablation [20]. These findings have led to several basic studies on quantifying the process of electrolysis through the use of transparent gels with pH dyes [12,21,22].

While minimally invasive thermal ablation is now commonly used in surgery “it has become common since the advent of modern imaging” [23]. Electrolysis is currently limited by the lack of an effective means to monitor the extent of tissue ablation deep in the body. The finding that pH changes are indicative of electrolytic tissue ablation [20], and the interesting results obtained with pH dyes marked gels [24–26], have suggested to us a way to monitor and image electrolysis. The pH fronts developing during the electrolysis process [6,24] are caused by evolution of protons (H^+^) and hydroxide (OH^-^) ions. While the relationship between pH level and electric conductivity is not straight-forward and depends on relative concentrations of other ions, the increased concentrations of protons and hydroxide ions would affect the local electrical conductivity of the tissue being electro-treated. The smaller relative sizes of the proton and the hydroxide ions could lead to a higher mobility compared to that of other ions typically found in biological solutions—we address this question further in the later discussion section. Historically, one of the foci of our lab’s work has been in the field of electroporation, i.e. the effect of applying brief pulses of high-magnitude electric field to a tissue [27,28]. The central hypothesis of this work is based on numerous empirical observations by our group of conductivity changes during electro-stimulation occurring even when the stimulation voltage was low and did not cause electroporation. We hypothesize in this work that the pH change induced changes in electric conductivity, could be used to detect, monitor or image the process of electrolysis deep in the body, in real time. It was further hypothesized that one possible technique to image electrolysis induced pH changes in real time, deep in the body, is Electrical Impedance Tomography (EIT). Electrical impedance tomography is used in a variety of scientific fields, from geology, to semiconductor characterization, to medical imaging. EIT produces an image of the electrical properties of the examined media. In a typical EIT application, electrodes are placed around the volume of interest, and small, sinusoidal currents are injected into the tissue, while voltages are measured on its boundary. Using the finite element method, the complex impedance of the analyzed domain is modeled, and a solution for the approximate impedance configuration that fits the measurements is obtained [29–31]. During the last four decades, substantial basic and applied research was done in the field of EIT. Our group has focused on applications for EIT in the context of monitoring minimally invasive surgery procedures such as cryosurgery [32], tissue viability [28,33] and electroporation [27],[34].

The primary goal of this study is to explore the hypothesis that changes in pH during electrolysis can be detected with EIT, for possible applications in monitoring tissue ablation with electrolysis. To explore the hypothesis we conduct an experimental study using a pH dye stained physiological saline agar-gel based phantom as a model for a living tissue, from an electrochemical standpoint. To investigate the hypothesis, we compare EIT reconstructed images to optical images acquired using pH-sensitive dyes embedded in the agar phantom that is exposed to electrolysis. In addition to validating the EIT-based approach using pH-sensitive dyes, we demonstrate a biological application of our EIT work by comparing a spatial map of bacterial viability exposed to electrolysis with the EIT image of the phantom during electrolytic treatment. Our results are promising, and invite further experimental explorations.

## Methods and Materials

### Tissue Model

**Fig 1:**
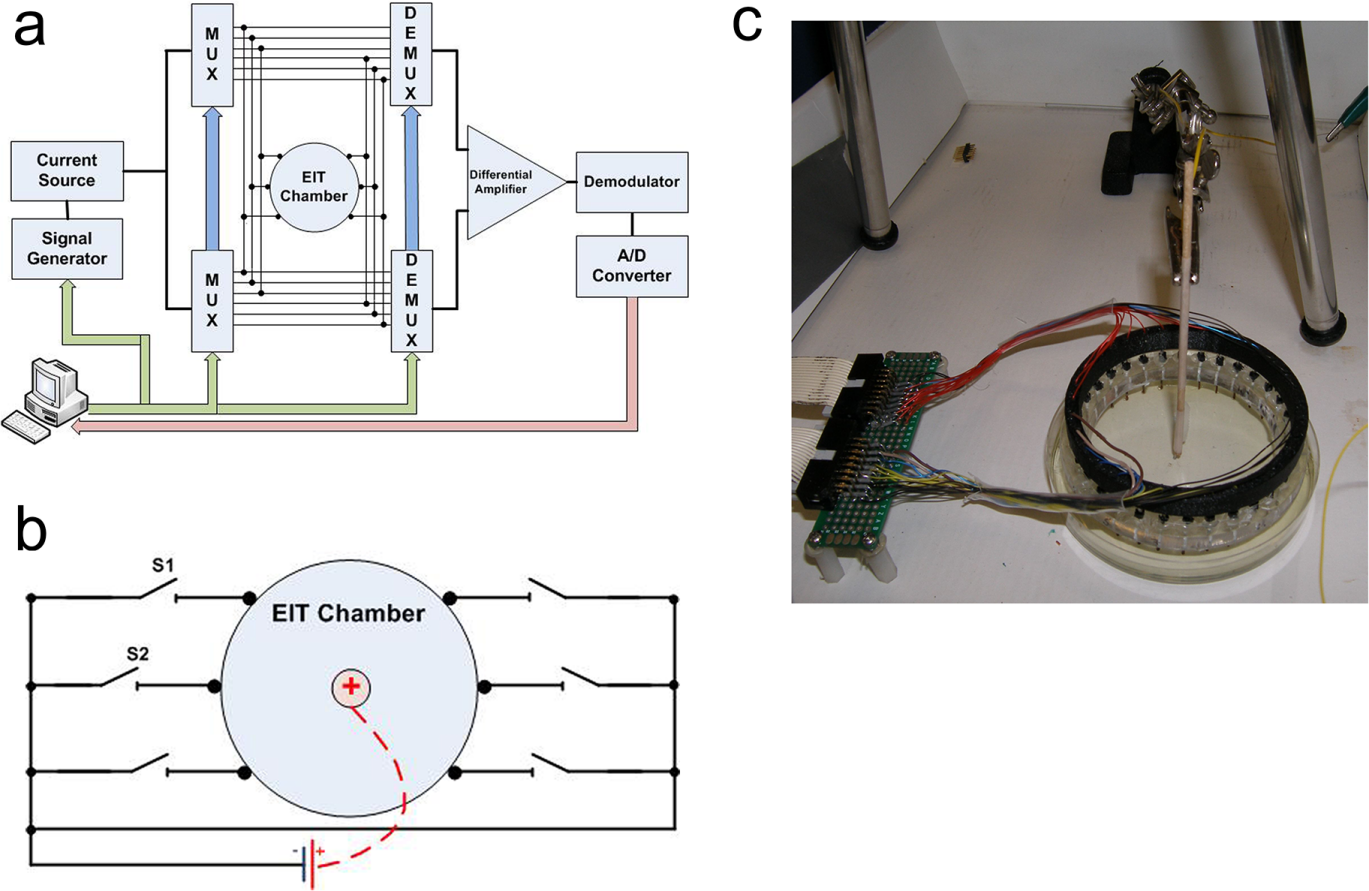
Electrical Impedance Tomography System (a) EIT System Schematic (reproduced from [29], MUX = multiplexer, DEMUX = demultiplexer) (b) Schematic of Experimental EIT Chamber for Electrolysis Experiments (c) EIT Chamber With Central Electrolysis Electrode in Our Experimental Setup.

Our tissue model consists of a physiological saline based agar gel phantom with electrical conductivity designed to simulate that of a tissue. To construct the phantom, 0.5% Bacto-Agar (Fisher Scientific) was mixed with 0.9 g/l Sodium Chloride (Fisher Scientific) in distilled water. The solution was then brought to a boil and poured into the Petri dishes. The conductivity of the agar phantom was measured to be approximately 0.14 *S*/*m* which is close to the range of hepatic tumor conductivity [35]. During the experiments, the EIT electrode holder was placed in the Petri dish with the electrodes galvanically coupled to the gel phantom (Fig 1).

### Experimental Model

To test the feasibility of EIT as a means to monitor the onset and extent of electrolysis in tissue, we have devised the following experiment: 1) A reference EIT image of the tissue phantom is taken, 2) Electrolytic stimulation is applied, 3) Another EIT reference image of the tissue phantom is taken. We leverage the differential nature of EIT images to represent the changes in conductivity, which are used as surrogates to regions of altered pH level. As a control study, we use pH sensitive dyes in order to estimate the boundary of the region where the pH has changed due to electrolysis. We use a digital camera (Casio Exilim EX-ZR100) to acquire optical images of the experimental chamber and correlate these images with the EIT reconstruction images. The results of several representative studies are presented in the following section: in each study we have repeated experimental steps 2) and 3), above, multiple times, in order to observe the evolution of the pH front over time.

### Bacterial Model

Lyophilized *E*.*Coli* of HB101 strain (BioRad) were grown in LB broth overnight and plated on LB broth based agar gel filled petri dishes. The LB broth for the overnight growth consisted of 1% BactoTryptone (BD), 0.5% Yeast Extract (BD), 1% NaCl (Sigma Aldrich) and 1.5% Agarose (Sigma Aldrich). For pouring the plates, we have held the sodium salt from the broth, in order to control the conductivity of the resulting gel. 6mm glass beads (Sigma Aldrich) were used for plating to ensure uniform coverage. After plating, the beads were removed and the plates were incubated for 15 minutes at 37°. The conductivity of the gel was measured around 0.2 *S*/*m*. At the experimental stage, the petri dish was separated from its lid and the EIT electrode array was lowered into the gel. On top of the EIT chamber, a 2 electrode holder with auxiliary electrodes was introduced into the gel. For the bacteria-focused experiments, only the auxiliary electrodes were used for stimulation, as opposed to the pH-sensitive dye experiments where we have also used the EIT electrodes for electrolytic stimulation. The stimulation sequence was applied using specified current and time parameters, with EIT snapshots being taken in the process as a monitoring step. The results section includes the exact current and time parameters used for each study. After the stimulation the petri dishes were covered and incubated for 24 hours. To evaluate viability we have visually inspected the petri dishes for areas where bacterial growth was inhibited.

### EIT Instrumentation

An EIT data acquisition system consists of a collection of electrodes, which are used to inject known sinusoidal AC current into the observed sample. Due to the sample’s conductivity, a potential develops on the sample. Due to the low frequency of the stimulation current, we ignore phase information and approximate impedance by Ohmic conductivity (resistivity). This potential is measured on the boundary using the electrodes not used for current injection. A schematic of a typical EIT system is presented in Fig 1A. In this work, we have used the EIT system described in [36], with *N* = 32 electrode surrounded circular chamber. We used an adjacent stimulation scheme [37], leading to each data set containing $\frac{N\cdot (N-3)}{2}=464$ independent measurements. While recent literature recommends against this stimulation scheme [38], the EIT system available to us in the lab has this stimulation scheme hardwired. While this clearly poses a limitation, and most likely leads to reduced image quality, we intend to address it in future work. After the data has been acquired, the data processing module of an EIT system attempts to reconstruct a conductivity map of the domain of interest from a set of known injected currents and measured resulting voltages, typically at the boundary of the geometric domain. In a typical EIT reconstruction algorithm, a map of impedance is guessed and the voltages resulting from injected currents calculated by solving Laplace equation in the domain. These voltages are compared to the measured voltage and the difference is then used as feedback for an iterative scheme. The guessed map of impedance is then updated, until the calculated and measured voltages agree within a certain tolerance. Here, we have used the EIDORS framework (v3.7.1) [39] with the complete electrode model (CEM). We modeled our electrodes as 2D needle electrodes surrounding a circular phantom area. The reconstruction was done by using a Gauss-Newton solver (EIDORS function inv_solve_diff_GN_one_step) with total variation (TV) regularization [40,41]. This approach works by attempting to minimize a cost function representing the overall voltage measurement discrepancy between the input (measured) voltages and the reconstruction algorithm’s internal model. The total variation functional is an attempt to regularize the reconstruction by making sure that the jumps in the final conductivity are bounded. The value of the regularization hyper-parameter was selected experimentally by taking a value that locally minimizes the residual error. This approach can be seen as a simplified version of the L-Curve method described in [42]. We used the value 0.003.

### Experimental Setup

The system is composed of 32 stainless steel electrodes mounted on a holder (Diameter = 75mm) lowered into a circular Petri dish (diameter = 85mm) chamber (Fig 1C). Following the work of [43] we have used internal electrodes in the experimental model to test the sensitivity limits of the system. The chamber contains the pH dye infused agar gel phantom which is imaged using EIT and optical digital camera. All the EIT stimulation currents had amplitude of 350μA and a frequency of 5kHz.

## Results and Discussion

### Anode Centered Experiment

In this experiment, we have placed a thin, stainless steel rod (diameter 0.6mm) in the center of the agar gel filled chamber. The central rod was connected using a copper wire to the positive terminal of the power supply and acted as the anode during this part of the experiment. For the cathode, all the 32 electrodes of the EIT were connected to each other by closing the switches *S*1.*S*32 presented schematically in the diagram on Fig 1B. The negative power supply terminal was then connected to the unified EIT electrodes. The EIT electrodes acted a distributed cathode in this case. As a control study we have employed two pH sensitive dyes: 1% phenolphthalein (Sigma-Aldrich) which turns pink/purple above pH 8.8 and acts as a basic indicator, and 2.4% pH indicator (Fresh water test-kit, API) which turns yellow at pH 6.0. Both pH indicators were added to the agar gel phantom before its solidification.

**Fig 2:**
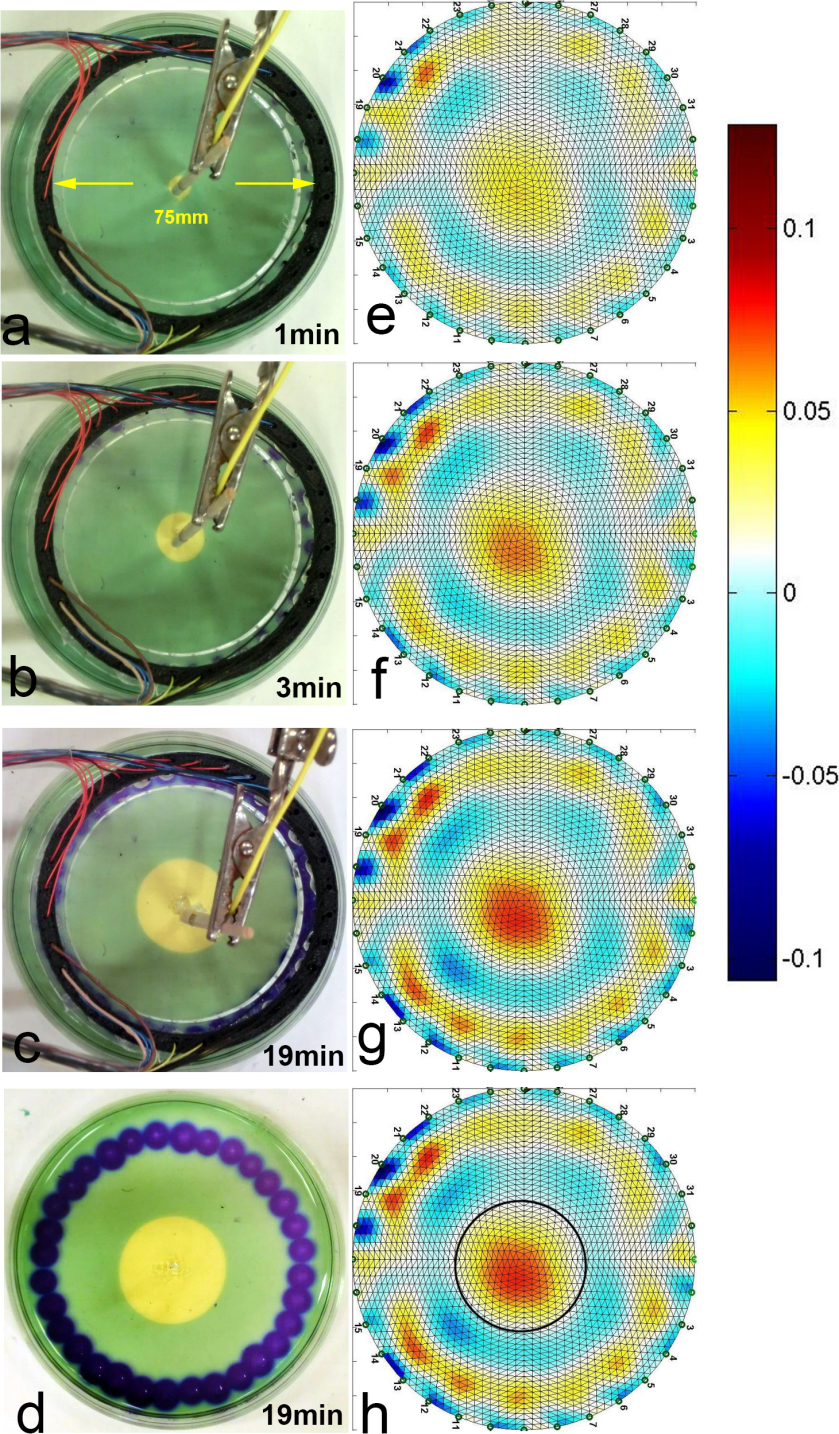
Anode Centered Electrolysis Experiment. EIT images: (a) After 1 minute, (b) After 3 minutes, (c) After 19 minutes, (d) After 19 minutes with overlaid outline of the pH altered region. Optical images: (1) After 1 minute, (2) After 3 minutes, (3) After 19 minutes (4) After 19 minutes with increased contrast

A photographic picture of our experimental chamber is presented in Fig 1C. The protocol of our experiment involved taking a control set of images: EIT and optical, before every electro-stimulation step. The electro-stimulation parameters including time and stimulation current are specified in Table 1. These parameters are typical to tissue ablation electrolytic processes, at the lower range of the parameters [4,44]. Fig 2 summarizes the results of our experiment by showing a sequence of image pairs: each EIT image is accompanied by its matching optical image which we used as a validation method. We have also excluded the possibility that the changes in impedance are caused by the pH-sensitive dye by running a control study where EIT data was acquired from agar gels without pH-sensitive dyes. The units of the color bars next to EIT images are specified in relative impedance changes i.e. 0.1 stands for 10% impedance change (increase for warmer colors and decrease for cooler ones).

**Table 1:** Summarizes the experimental parameters used in this work.

Experiment	Current	Total Charge	Time
Anode centered	1ma	1.14C	19min
Cathode centered	1ma	2.16C	36min
Two electrodes	2ma	1.44C	12 min
Bacterial viability	2ma	5.4C	45min

The current was delivered at 1*mA*, and the delivered charge dosage was 1.14*C*, which falls within a range of a typical electro-chemo therapy stimulation charge dosage [4,44]. Fig 2E— 2G show the EIT images at selected time points whereas Fig 2A— 2C show the corresponding optical images. Fig 2H shows the final result of the gel model after the EIT electrodes have been removed. It can be seen that the EIT images of the pH from near the central electrodes are in good correspondence with the pH indicator dye: the central spot around the anode grows over time in both the optical and the EIT images. The data shows a good qualitative correspondence between the EIT reconstructed images and their optical counterparts. We have chosen to include representative images corresponding to times t = 1 minutes, t = 3 minutes and t = 19 minutes. The contrast of the image in Fig 2H was increased to show the altered pH indicator at the perimeter, close to the distributed anode.

The color-bar presented to the right of the figure helps interpret the EIT results: the EIT images are taken in differential mode which means that the images show differences relative to a reference image taken before any electrolytic stimulation was applied. Warmer colors correspond to increased conductivity while colder colors correspond to decreased conductivity in the sample.

The change in conductivity in the center of the gel phantom captured by the EIT system can be explained by noting the relatively small radii of protons (*H* ^+^, 0.88*fm*) compared to the radii of other physiological ions such as chlorine ions (Cl^-^ 167 *pm*) and Na^+^(116 *pm*). The smaller ions produced in the electrolytic reactions led to increased local mobility which in turn led to increased conductivity around the anode. While the relationship between pH and change in conductivity depends on multiple factors including the concentrations of various ions and their respective mobilities, the results indicate an observable correlation between changes in pH and changes in conductivity. To facilitate the comparison of results, we superimposed the margin of the altered region in the pH dye infused gel onto the EIT image in Fig 2H. The margin of the marked region indicates the boundary of the highest pH of 6. For our stimulation conditions, the area with altered pH takes a circular shape. The region within the marked boundary corresponds to pH range of 1–6. Our future work is envisioned to explore the relationship between pH and the electric conductivity. To control for confounding impedance changes we have measured changes in temperature during the electrolytic stimulation, and no observable temperature change was detected. In our previous work with higher current densities we have observed electro-osmotic effects [45], which could further contribute to the changes in impedance, but under the current stimulation regimes, these effects were not observable in this experiment. Characterizing the impact of electro-osmosis on changes in impedance is another research direction which we chose to focus on in future explorations.

### Cathode Centered Experiment

**Fig 3:**
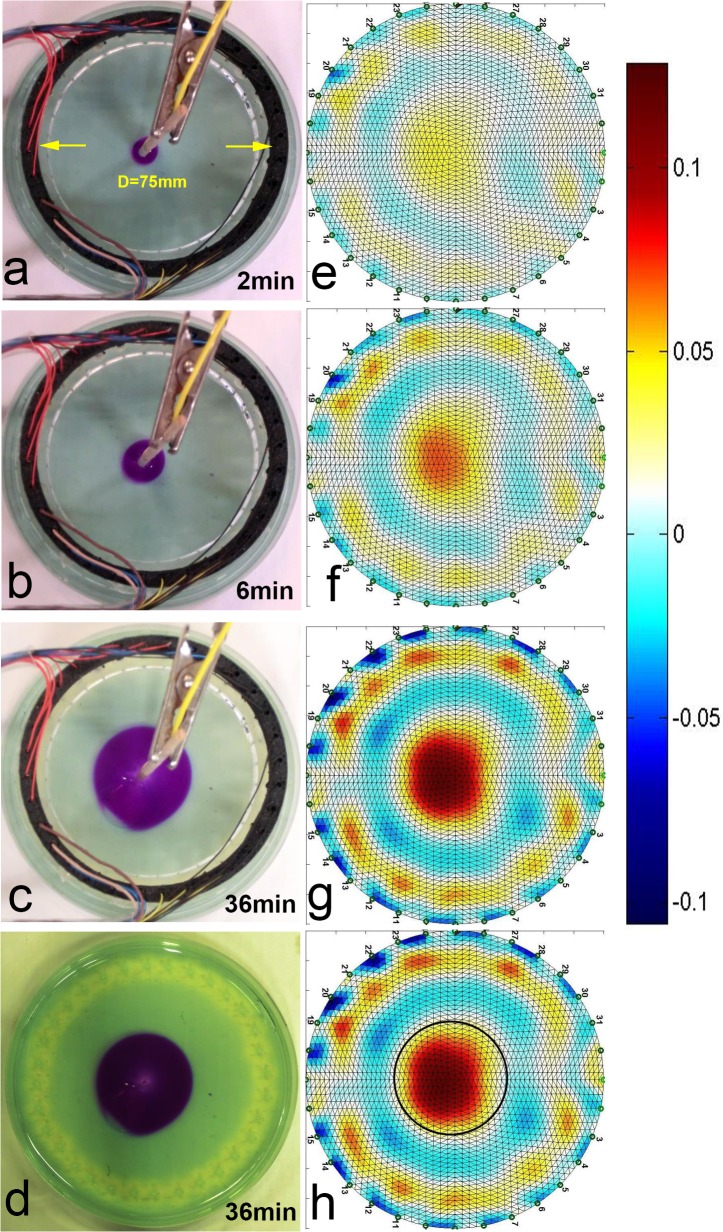
Cathode Centered Electrolysis Experiment. EIT images: (a) After 2 minute, (b) After 6 minutes, (c) After 36 minutes (d) After 36 minutes with overlaid outline of the pH altered region. Optical images: (1) After 2 minute, (2) After 6 minutes, (3) After 36 minutes (4) After 36 minutes with increased contrast

In this part of the experiment, we have reversed the roles of the anode and the cathode. The same pH indicators were used as before: 1% phenolphthalein which turns pink/purple above pH 8.8 and acts as a basic indicator, and 2.4% pH indicator (Fresh water test-kit, API) which turns yellow at pH 6.0. As in the previous section, both pH indicators were added to the agar gel phantom before its solidification. The protocol of this experiment involved taking another control set of images: EIT and optical, before every electro-stimulation step. The electro-stimulation parameters including the current and the stimulation duration are specified in Table 1. Fig 3 presents the results of the experiment by showing a sequence of image pairs: each EIT image is accompanied by its matching optical image which we used as a validation method. The units of the color bars next to EIT images are specified in relative impedance changes i.e. 0.1 stands for 10% impedance change (increase for warmer colors and decrease for cooler ones). The overall charge dosage was charge dosage was 2.16*C*. While this dosage falls within a range of a typical electro-chemo therapy procedure, it is a larger charge dosage compared to the anode centered experiment. We have administered more charge in the cathode-centric experiment because the altered pH front indicated by the pH-sensitive dye (phenolphthalein) was growing slower in the cathode-centered case. A possible explanation to this difference is the relative size of the *H* ^+^ and the *OH* ^−^ ions, and we discuss this discrepancy in more details in a later section (*Bacterial Sterilization Model*). Fig 3E –3G show the EIT images at selected time points whereas Fig 3A— 3C show the corresponding optical images. Fig 3D shows the final result of the gel model after the EIT electrodes have been removed. It can be seen that the EIT images are in good correspondence with the pH indicator dye: the central spot around the cathode grows over time in both the optical and the EIT images. Moreover, while it is too subtle to see in the optical images, the EIT imaging clearly shows a circular feature at the periphery of the EIT chamber. This peripheral region with reduced pH level can be clearly distinguished in Fig 3D by its distinguished yellowish color. It can only be seen after the EIT electrodes have been removed. The data shows a good qualitative correspondence between the EIT reconstructed images and their optical counterparts. We have chosen to include representative images corresponding to times t = 2 minutes, t = 6 minutes and t = 36 minutes. An accumulation of liquid, presumed to be water can be observed around the cathode in the form of a growing bubble. We have attributed it to the osmotic effects of electrolysis reported by other researchers. Fig 3D shows the optical image after the EIT electrodes have been lifted at the end of the experiment. The contrast of the image in Fig 3D was increased to show the altered pH indicator at the perimeter, close to the distributed anode.

The change in conductivity in the center of the gel phantom captured by the EIT system can be explained by noting the relatively small radii of hydroxyl ions (*OH* ^−^, 110*pm*) compared to the radii of other physiological ions such as chlorine ions (Cl^-^ 167 *pm*) and Na^+^(116 *pm*). The smaller ions produced in the electrolytic reactions led to increased local mobility which in turn led to increased conductivity around the cathode. This behavior is similar to the anodic case, although the degree of change in conductivity is different, possibly due to the difference in relative sizes of protons and hydroxyl ions. To facilitate the comparison of results, we superimposed the margin of the altered region in the pH dye infused gel onto the EIT image in Fig 3H. The margin of the marked region indicates the minimal pH of 8.8. For our stimulation conditions, the area with altered pH takes a circular shape. The region within the marked boundary corresponds to pH range of 8.8–14. Like in the previous experiment, to control for confounding impedance changes we have measured changes in temperature during the electrolytic stimulation, and no observable temperature change was detected. In our previous work with higher current densities we have observed electro-osmotic effects which could further contribute to the changes in impedance, but under the current stimulation regimes, these effects were not observable in this experiment.

### Two Internal Electrodes Experiment

In this part of the experiment, instead of using the EIT electrodes as a distributed electrode, we have utilized two graphite electrodes made of pencil lead (Pentel super HB 0.7mm). The electrodes, mounted in a horizontal holder were placed perpendicularly to the gel phantom. The electrodes were inserted 5mm deep into the gel. We have used a 5% pH indicator (RC Hagen wide range). As in the previous experiments, the pH indicator was added to the agar gel phantom before its solidification.

**Fig 4:**
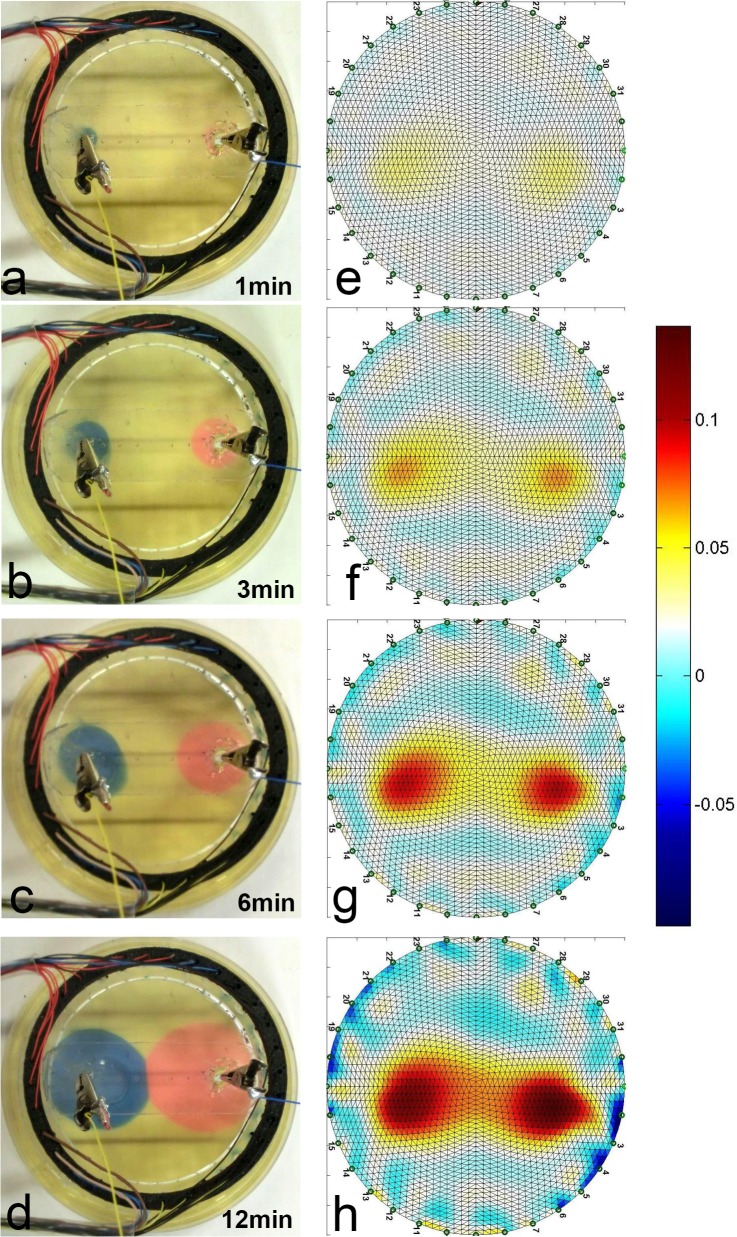
Two Electrodes Electrolysis Experiment. EIT images: (a) After 1 minute, (b) After 3 minutes, (c) After 6 minutes, (d) After 12 minutes. Optical images: (1) After 1 minute, (2) After 3 minutes, (3) After 6 minutes (4) After 12 minutes.

The protocol of this experiment involved taking another control set of images: EIT and optical, before every electro-stimulation step. The electro-stimulation included a sequence of direct current injections at 2mA of the following durations: [1min, 1min, 1min, 1min, 1min, 1min, 1min, 5min]. Fig 4 presents the results of the experiment by showing a sequence of image pairs: each EIT image is accompanied by its matching optical image which we used as a validation method. The units of the color bars next to EIT images are specified in relative impedance changes i.e. 0.1 stands for 10% impedance change (increase for warmer colors and decrease for cooler ones). The delivered charge dosage was 1.44*C* which falls within a range of a typical electro-chemo therapy stimulation charge dosage [4,44]. Fig 4E— 4G show the EIT images at selected time points whereas Fig 4A— 4C show the corresponding optical images. It can be seen that the EIT images are in good correspondence with the pH indicator dye: the central spot around the anode (red) grows over time in both the optical and the EIT images, and the same is observed for the spot around the cathode (blue). We have chosen to include representative images corresponding to times t = 1 minutes, t = 3 minutes, t = 6 minutes and t = 12 minutes. It is notable that both the optical and the EIT approaches are able to image the collision of the basic and the acidic fronts (Fig 4H and 4D).

As in the previous two experiments, to control for confounding impedance changes we have measured changes in temperature during the electrolytic stimulation, and no observable temperature change was detected. In our previous work with higher current densities we have observed electro-osmotic effects which could further contribute to the changes in impedance, but under the current stimulation regimes, these effects were not observable in this experiment, even though the currents were higher than in the case with one central electrode.

### Bacterial Sterilization Model

**Fig 5:**
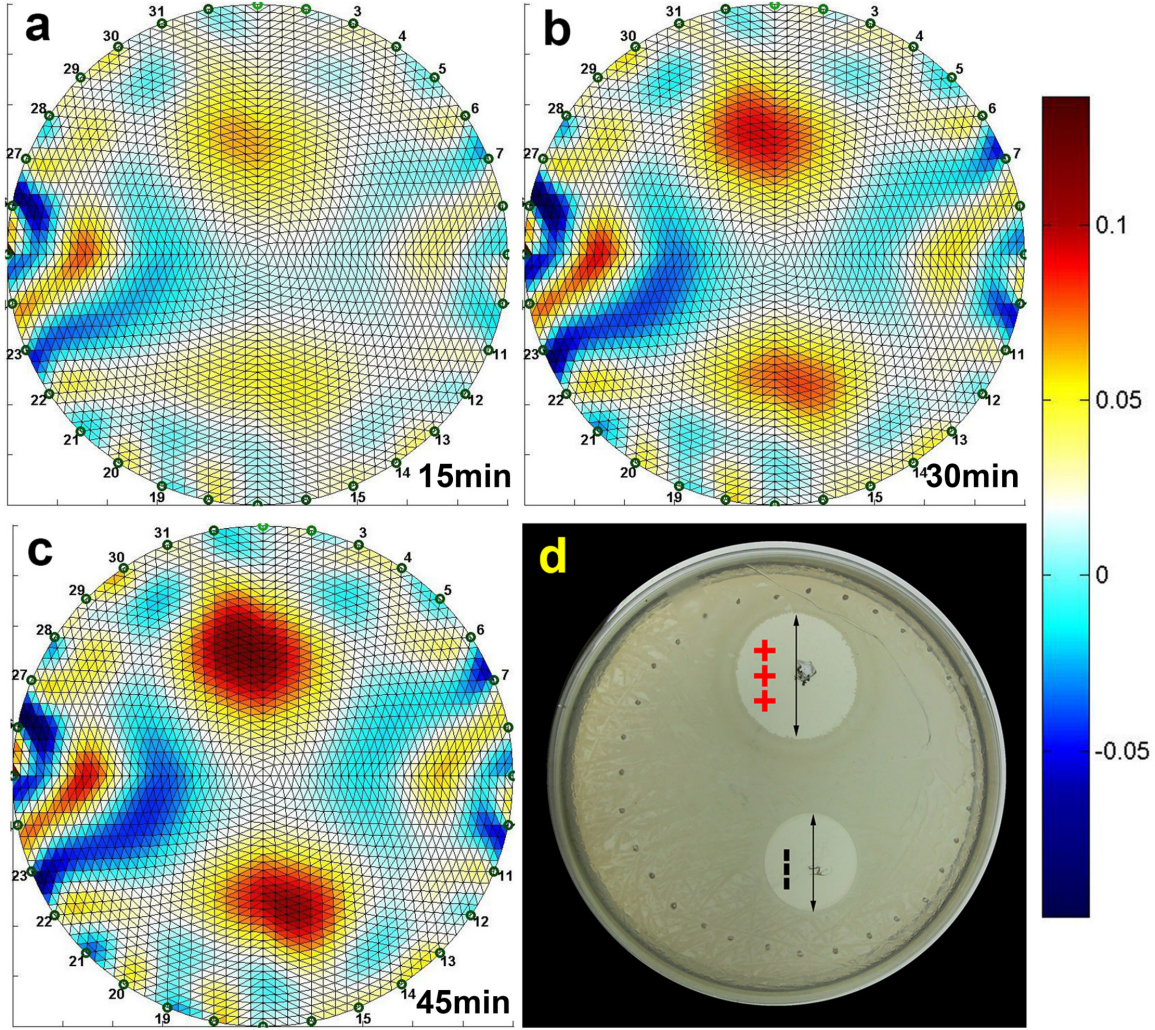
Bacterial Viability Experiment. EIT images: (a) After 15 minutes, (b) After 30 minutes, (c) After 45 minutes. (d) Optical image of growth patterns after 24 hour incubation

To confirm the efficacy of our method in a biological model, we have used EIT for imaging electrolysis in an agar dish plated with *E*.*Coli* bacteria. The liquid bacterial culture was first plated as described in our methods, and then a current of 2mA was administered using the auxiliary electrodes. The total administered charge dosage was 5.4*C* which falls within a range of a typically delivered charge during an electro-chemo therapy stimulation[4,44]. Fig 5 shows a comparison between the EIT imaging data and a bacterial viability pattern captured using an optical, digital camera after 24 hour growth period. The units of the color bars next to EIT images are specified in relative impedance changes i.e. 0.1 stands for 10% impedance change (increase for warmer colors and decrease for cooler ones). Fig 5A–5C indicate two growing regions of increased conductivity, around the auxiliary electrodes through which electrolytic stimulation was applied. We have chosen to include representative images corresponding to times t = 15 minutes, t = 30 minutes and t = 45 minutes. Fig 5D shows the optical image of the viability pattern taken 24 hours post-stimulation. It is interesting to note that both the EIT images as well as the optical image exhibit asymmetry with regards to the anodic and the cathodic regions under our experimental conditions. The brighter upper spots in the EIT images, in particular in the one shown in Fig 5C indicates that the conductivity of the anodic region has changed to a larger degree than the conductivity of the cathodic region. This discrepancy can be attributed to the relative radii of protons (*H* ^+^, 0.88*fm*) and hydroxide ions (*OH* ^−^, 110*pm*). Due to their relative smaller size, the protons are more mobile hence contributing to a larger extent to the conductivity increase around the anode. The increased mobility causes the bactericidal pH region around the anode to be larger than around the cathode. This is supported by the viability observations presented in Fig 5D. To clarify, the circular pattern of dots around the bacterial culture dish corresponds to the EIT electrodes imprinted in the gel when the EIT chamber was lowered.

### Limitations and Future Work

While our results show a degree of correlation between EIT measurements and optical measurements obtained using pH-sensitive dyes, several key questions arise which need to be answered before EIT can be successfully used in clinic as an imaging technique for electrolysis. These include inquiries into the nature of the relationship between pH and conductivity, or the differential changes around the cathodic and the anodic regions. Since other imaging techniques for electrolysis were not reported in the literature, our current benchmark is optical pH-sensitive dye images, but other imaging modalities such as MRI might potentially result in a better comparison standard. There are several limitations inherent to our work: first, our tissue model is limited to an agar gel based phantom which is a simplistic way to represent a biological tissue. Further experiments are envisioned with living tissues replacing the gel phantom. In addition to the inherent limitation of the model, the EIT system we have employed in this work is outdated and was used as a first order, crude approximation in order to establish if further experimental effort can be justified. Clearly, optimizing EIT acquisition and reconstruction parameters would result in better image quality which might increase the likelihood of developing a clinically viable imaging modality. Assuming that the method works on a living tissue, it could potentially be applied in a clinical setting: in this scenario EIT might become a monitoring method for electrolytic ablation in the treatment of breast cancer, see e.g. [46]. This could allow a more controlled real-time monitored treatment at a low cost, which could provide a new set of medical tools to resource constrained communities. The key limitations are foreseen to be the tissue types where the therapy could be applied. For example, when the tissue exhibits high baseline conductivity, the changes in pH might not affect the conductivity to such an extent that can be detected using an off-the-shelf EIT system. In a highly conductive tissue, the pH changes can fall under the noise margin of EIT measurements. We have limited this work to conductivity corresponding to a hepatic tumor, in which detection feasibility was demonstrated. We intend to explore the relationship between pH and conductivity, which will allow us to characterize the conductivity regions in which our approach could be potentially useful.

Demonstrating the proof of concept for a new application of the EIT technology was the main goal of this study, i.e. our purpose was to show that EIT is sensitive enough to image electrolysis and we do not claim any improvements in the technology itself. We believe that using EIT for this new application, i.e. monitoring electrolysis could become an enabling technology for planning and monitoring low-cost minimally invasive surgery procedures involving electrolysis. The configuration employed in this study and the *E*.*Coli* experiments have an immediate application in relation to the use of electrolysis and electrolytic products to sterilize wounds and destroy microorganisms on the surface of the body, the skin [1,47]. Placing an EIT array around a surface wound, treated with electrolysis, could provide a means to monitor the treatment. While EIT has numerous technical limitations, the application in the configuration tested which focuses on the outer surface of the body is less restricted by the typical constraints of EIT. The microorganism study employed here is directly relevant and illustrative of this application. Another field of application is in relation to the combination between reversible electroporation and electrolysis[25,26,48,49]. We have recently shown that generating electrolytic products, prior, during or after reversible electroporation can be seen as a new method for tissue ablation[48,49]. The ability to monitor the extent of electrolysis could provide greater control over tissue ablation with this combination, with possible applications to treatment of melanomas. Reversible electroporation combination with bleomycin is a tissue ablation technology known as electrochemotherapy, also used primarily for treatment of melanoma [50]. Reversible electroporation is also an important technology for gene vaccination[51]. However, it was recently found that electrolytic products can be generated during reversible electroporation for insertion of genes into cells. These products may cause cell death and be detrimental to gene therapy protocols[25]. Using EIT to detect the production of electrolysis during reversible electroporation may benefit gene vaccination; in particular in configurations in which the electroporation is near the surface of the body, the skin.

Given the scope of our work, we see how it suffers from the generic limitations of EIT i.e. nonlinear current spread and the uncertainty associated with the position of the electrodes. Having said that, these are challenges that are not unique to the application we suggest and we do not claim to solve them in this paper. Furthermore, as discussed earlier, the configuration analyzed here may be less susceptible. Our wish in this work was to demonstrate that EIT can reliably detect pH changes. We see this work as a first step towards developing a novel imaging approach and this paper was focused on validating the fundamental feasibility of the method.

### Conclusion

In summary, we report experimental findings that support the hypothesis that electrolysis induced pH-changes lead to local conductivity changes in a physiological gel tissue model. It is these changes in conductivity that can be captured in real time by EIT. Our results indicate the feasibility of using EIT as a means to monitor dynamic changes in local pH level of a biological sample during an electrolysis process. Our work uses agar-based gel model with conductivity in the range of a biological tissue, and is validated vs. optical images utilizing pH indicator dyes. In addition we demonstrate the relevance of our work in the biological context by correlating bacterial viability data with EIT measurements. Our results are promising, and invite further experimental explorations.
